# Pathways from media attention and peer communication to body dissatisfaction: the moderating role of protective filtering

**DOI:** 10.1186/s40359-023-01491-x

**Published:** 2023-12-20

**Authors:** Jing Ji, Xiaoli Xiang, Ren Chen, Zenghong Chen, Jing Yan

**Affiliations:** 1 School of Health Service Management, Anhui Medical University, Hefei, 230032 China; 2 Department of Ophthalmology, The Affiliated Changshu Hospital of Nantong University, Changshu, 215500 China; 3 Department of Plastic surgery, The second Hospital of Anhui Medical University, Hefei, 230601 China

**Keywords:** Body dissatisfaction, Media attention, Peer communication, Protective filtering, Body surveillance

## Abstract

**Background:**

Negative body image is a common psychological phenomenon among young Chinese women, and merits further investigation. Peers and the media are important factors that associated with body image. This study explored how media and peers promote body dissatisfaction among young Chinese women, including the mediating role of body surveillance and the moderating role of protective filtering.

**Methods:**

3499 women from the general China community aged 18–40 years (M = 23.44 years, SD = 1.18 years) were investigated with sociocultural attitudes towards appearance scale-3, objectified body consciousness scale and protective filtering scale. The data were analyzed by using a moderated mediation model with SPSS and the Process 4.0 macro.

**Results:**

Correlational analysis results indicated that body surveillance acted as a chained indirect effect between the internalization of media information and body dissatisfaction, as well as between peer comparison and body dissatisfaction. Moreover, protective filtering was demonstrated to moderate the path of media attention affecting the internalization of media information and the path of peer communication affecting peer comparisons.

**Conclusion:**

Our results contribute to the understanding of the sociocultural mechanisms underlying young women’s negative body image. Furthermore, investigating the moderating effect of protective filtering is conducive to guiding future female positive body image interventions.

## Introduction

Body dissatisfaction is a body image evaluation attitude characterized by negative self-perception about body, including body size, weight, and attractiveness [1]. Social media is full of idealized and sexualized portray of women’s bodies. Compared to male groups of all ages, young adult women pay more attention to ideal beauty-related photos, videos and messages on social media, and body dissatisfaction is a pervasive problem among young adult women [2, 3]. People’s attitude towards their own bodies not only affects their cognition but also their behavior. Body dissatisfaction can lead to a range of harms such as psychological distress, low self-esteem, and eating disorders [4, 5]. Most studies have focused on the negative consequences of body dissatisfaction rather than its antecedents [6, 7]. In addition, few studies have focused on the moderating effect of certain protective factors on negative body image from an information dissemination perspective.

The tripartite influence model of social culture theory is an important theory to interpret female body image which proposes that sociocultural factors affect individuals’ satisfaction with their physical appearance [8]. This crucial model has been used to investigate individuals’ body image and the negative distress caused by body dissatisfaction. Furthermore, a substantial body of research literature has proven that three main factors of the tripartite influence model (social media, peers, and parents) affect women’s negative body image through two mechanisms: internalization of the beauty-ideal and body comparison processes [3].

However, the tripartite influence model does not elaborate how internalization and comparison processes affect body dissatisfaction. The self-objectification theory emphasizes that when women are constantly exposed to information about ideal beauty of social media and negative comments on their bodies from peers, the process of self-objectification will be initiated. They examine their bodies from the perspective of observers. Such continuous monitoring of their bodies will eventually lead to body dissatisfaction and shame [9]. Meanwhile, peer influence, especially peer conversations, would stimulate appearance comparison among peers, subsequently affecting body dissatisfaction [10, 11].

In addition, the latest research proposes that protective filtering (Women process information in a self-protective manner, internalizing most positive body image messages while rejecting and reconstructing most negative body image messages) is a protective factor against body dissatisfaction [4], thereby protecting and promoting body satisfaction among women. The term “protective filtering” was first coined by Wood-Barcalow et al. [11] based on their qualitative investigation of the characteristics of women with a positive body image. Protective filtering is defined as, “accepting information that is consistent with positive body image while rejecting messages that could endanger it”. That is to say, it is an information processing strategy conducive to the construction of positive body image and help women defend themselves against the negative effects of negative external information on body image.

Although these theories and research findings were initially developed and used in a Western socio-cultural context, it has also proved applicable in the Chinese cultural background [12]. In China, collectivism spirit is rooted in the social environment, and individuals tend to feel more social pressure to care about others judgments and thoughts [13, 14]. Therefore, Chinese young adult women may easily be influenced by mass media and interpersonal relationships. For social media regulators, parents, and Chinese young adult woman, it is essential to understand the components of women’s body dissatisfaction. Based on the theory of the tripartite influence model of sociocultural theory, objectification theory, and previous literature on protective filtering, this study developed a hypothesized framework to better understand how media attention to appearance-related messages, peer conversation, and protective filtering influences body dissatisfaction. Particular attention was given to the role of protective filtering.

## Theoretical framework and hypothesis development

### The tripartite influence model

The tripartite influence model is commonly used by scholars to explain how body dissatisfaction is formed under the influence of participation in photo-related social media platforms and peer interactions [15]. The main point of this model is that three aspects that affect body image (media, parent, and peers) including two primary mechanisms (internalization of the beauty-ideal and appearance comparison). Internalization is one of the focuses of the tripartite influence model, which means that individuals’ values and social standards are affected by social culture and social standards as their norms of behavior [16, 17]. The media, especially social media, is a powerful driver of transmitting sociocultural beauty standards and expectations, such as ideal size, weight, and fashion. With its rapid development, the number of young adult women paying attention to information promoting the “ideal body image” is rapidly increasing. Media attention refers to exposure to or use of certain media types, most commonly television, newspaper, the Internet, or social media. Slater et al [18]. explained it as “people’ s tendency to devote cognitive effort to particular types of media messages consciously”. In the field of body image, media attention is often behaviorally manifested by spending excessive time and energy on social media related to body image, as well as actively following and searching for information related to “ideal beauty” on the internet [17, 19]. Accumulating evidences suggest that excessive attention to the idealized societal standards of beauty created by social media may affect how individuals process body-related information, making it easier for them to form negative body images [20, 21].

Meanwhile, a person’s values and self-image are internalized through the subtle influence of parents and significant others [22]. Research has shown that women’s attitudes towards their bodies are influenced by their parents’ memory of emotional indifference [23]. However, as women reach adulthood, they have less contact with their parents and more contact with their peers, and the influence of peers gradually strengthens. Peer communication refers to talking and discussing with peers about body image such as appearance, image and attractiveness [24]. These appearance-related communication provides an environment in which image concerns are focused upon, interpreted and subsequently come to be valued. Peer conversations about appearance are common in the daily lives of young adult women indicating that individuals focus on body image in the process of interpersonal communication. Several studies have found that when women talk more frequently about their appearance with their friends, they also have an increased sense of comparison about their bodies [25, 26]. Furthermore, when discussing their bodies with friends, women tend to evaluate their appearance by comparing themselves with others, which is relevant to more serious negative body image and disordered eating [27]. Based on aforementioned viewpoints, we hypothesize:

H1: Media attention is positively associated with the internalization of media information.

H2: Peer communication is positively correlated with peer comparison.

### The mediating role of body surveillance

Objectification theory indicates that self-objectification is the process by which women treat themselves as objects to be evaluated and internalize others’ evaluations of their own bodies [9], which has been proven to be associated with increased anxiety and dissatisfaction with their bodies [28, 29]. Body surveillance is a behavioral manifestation of self-objectification and is commonly used to reflect the level of self-objectification [30, 31]. Women with a higher level of self-objectification spend more time monitoring their body (i.e., body surveillance) to ensure that they conform to the ideal of social beauty [29]. The extent of body surveillance has been shown to positively correlate with the level of body dissatisfaction [32].

In addition, objectification experiences include not only women’s internalization of sexualized information conveyed by social media, but also interaction and commentary with peers about their own and others’ body appearance. On the one hand, social media often focuses on women’s body in a sexualized way, which tends to standardize the aesthetics of women’s appearance [33]. Previous studies proved that once young adult women internalize these standards, it may trigger body surveillance to monitor how one’s body is being evaluated by others, which then lead to negative psychological outcomes or perception of flaws in one’s appearance, producing body dissatisfaction [34]. On the other hand, interaction and commentary with peers about their own and others’ body appearance reinforce the intensity of body image comparisons between young adult women and their peers, which subsequently triggers body surveillance. Wang et al. [33] posited that body surveillance mediated the relationship between appearance-relevant comparison and body shame. Based on the above views, we posit the following hypotheses:

H3a: Body surveillance mediates the relationship between internalization of media information and body dissatisfaction.

H3b: Body surveillance mediates the relationship between peer comparison and body dissatisfaction.

### The moderating role of protective filtering

Protective filtering was first proposed as an intervention strategy that incorporates multiple features of positive body image in a qualitative study, and is thought to help focus women’s body investment on self-care and functionality and preserve their positive body evaluation [11]. When exposed to information related to appearance in social media and direct social environment, protective filtering refers to accepting positive information that is benefit to women’s body appreciation, while rejected or reconstructed negative information [35]. That is to say, selectively filtering in positive source information and counteracting negative source information help promote and maintain their positive body evaluation [36, 37]. For example, Andrew et al. [38] revealed that women with protective filtering strategy after viewing slim female images on social media did not compare themselves with the beauty-ideal or experience a negative change in their satisfaction with their bodies.

Furthermore, in prior body image studies [35, 39], protective filtering was proved to be an effective intervention to lower women’s internalization of beauty-ideal and body depression. Based on fertile previous studies, it can be supposed that protective filtering can moderate the effects of external information (information from social media and peer communication) on internal cognition (such as internalization and peer comparison). Following these statements, we hypothesize:

H4a: Protective filtering moderates the effects of media attention on the internalization of media information.

H4b: Protective filtering moderates the effects of peer communication on peer comparison.

**Fig. 1:**
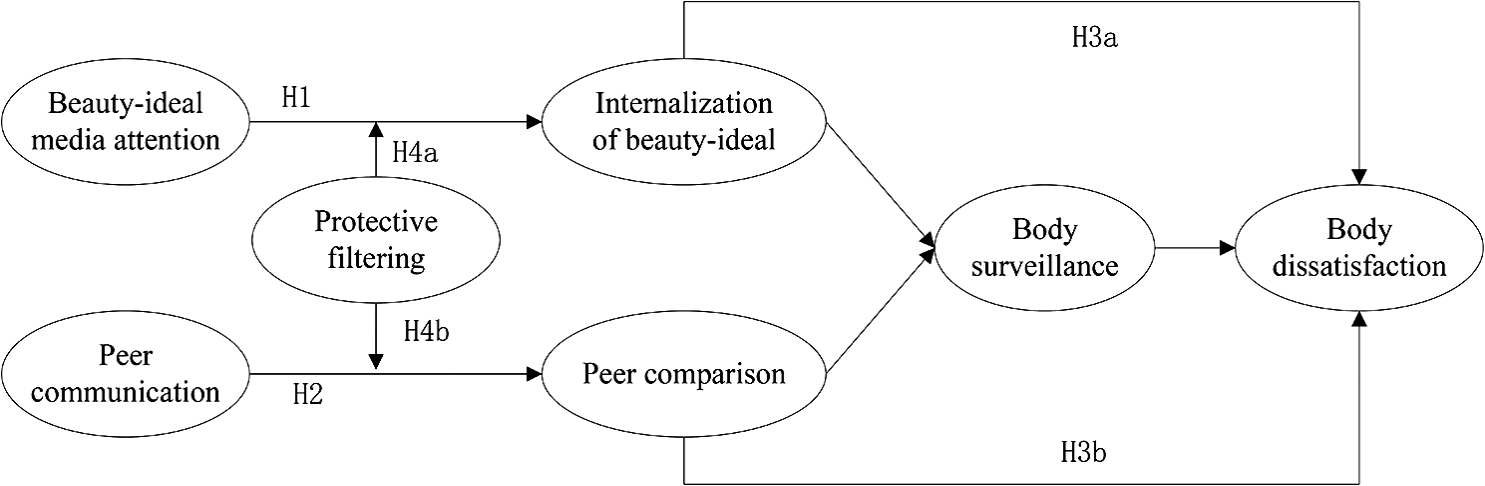
Hypothesized model

Based on the aforementioned hypotheses, the research framework is depicted in Fig. 1.

## Method

### Participants

Participants were recruited through Sojump, a questionnaire collection platform contained more than 260 million registered users in mainland China, which provides functions equivalent to Qualtrics [37] and is widely used by many Chinese researchers to conduct online surveys [40]. Since the subjects of this study were mainly women and men were not included in the study, we randomly sent the survey link and brief introduction to female registered users of Sojump via email. We obtained the informed consent of the participants through a consent page on the first page of online questionnaire, and assured them that the questionnaire contained no identifying information to ensure confidentiality. After agreeing to participate in the survey, participants began to fill out the questionnaire in a self-administered manner. Data collection consisted of two parts: a demographic survey and a series of body image questionnaires. In the demographic survey section, participants were asked to indicate their age, marital status, and educational background. After completing the questionnaire survey, all participants have received a cash reward of CNY 5 (equivalent to USD 0.7) online in exchange for their participation.

The online survey lasted three months (March 4 to May 15, 2022). In the end, a total of 4,057 questionnaires were completed. 558 questionnaires were declared invalid based on three criteria: missing data, uniform responses for all questions, and the participants’ fill-in time (less than five minutes is assessed to be unqualified). Finally, 3,499 valid questionnaires were obtained, yielding a valid response rate of 86.2%. All participants were from the general China community, aged 18–40 years (M = 23.44 years, SD = 1.18 years), and the great majority of participants (62.2%) had a college degree or higher.

### Outcome measures

#### Beauty-Ideal Media attention and internalization of the beauty-ideal scale

We used the Sociocultural Attitudes Towards Appearance Questionnaire-3 [41](SATAQ-3) to assess the extent of media attention and internalization of the beauty ideal; the scale contains two subscales: Media Attention Subscale (including six questions, such as, “Social media is an important source for me to get information on fashion, beauty, weight loss, and more”) and Internalization Subscale (including nine questions, such as, “When I see photos of other people on social media, I compare my appearance or body to them”). The response format was a 5-point Likert scale ranging from I completely disagree (= 1) to I completely agree (= 5), higher scores in the scale reflect higher levels of media attention and information internalization. Cronbach’s α was 0.89.

#### Peer communication and peer comparison scale

Frequency of peer communication and peer comparison were assessed with the Peer Influence Scale drawn from the SATAQ-3 [41], which contains six items and is divided into two subscales: Peer communication scale (three questions, such as “I often talk to my friends about physical appearance”) and information internalization scale (three problems, such as “When I see pictures posted by my friends, I compare myself to them”). The response format was a 7-point Likert scale ranging from I completely disagree (= 1) to I completely agree (= 7), higher scores in the scale reflect higher degree of peer communication and information internalization. Cronbach’s α was 0.88.

#### Objectification body awareness scale-body surveillance subscale

Frequency of body surveillance was assessed with the Objectified Body Consciousness Scale (OBCS-Body Surveillance) drawn from the Body Surveillance Scale [42], which contains eight items (e.g., “When I look in the mirror before I go out, I am often dissatisfied with how I look in the mirror”), and use the 5-point Likert scoring method (1 = completely disagree, 5 = completely agree), higher scores in the scale reflect body surveillance. Cronbach’s α was 0.86.

#### Protective filtering scale

Items to measure protective filtering were newly developed. Although protective filtering is an important construct for examining how individuals process and respond to appearance-related information, body image research lacks a professional quantitative protective filtering scale. Thus, the present study measured protective filtering with five items, which were modified from the work of Ornella et al. [4] and Tylka and Wood-Barcalow [11]. The first three items (such as “I accept information from social media that encourage women to be themselves and make their own body image”, “I often actively block messages, photos and videos that make me anxious about my appearance, " and “I try to relate the ideas in the body-related information or comments to my health”) were from Ornella et al. [4, 43], which were used to investigate women’s information permitting. The last two items (such as “I don’t pay too much attention to information about ideal beauty on the Internet”, and “I ignore negative body-related information”) were from the Tylka and Wood-Barcalow [11], which were used to measure women’s information forefending or blocking out. The response format was a 7-point Likert scale ranging from I completely disagree (= 1) to I completely agree (= 7). The average score of each question was counted as the total score of protective filtering, higher scores in the scale reflect higher extent of protective filtering. Cronbach’s α was 0.86.

#### Body dissatisfaction scale

Frequency of body dissatisfaction was assessed with the Body dissatisfaction Scale in the SATAQ-3 [41]. The scale consists of eight items, for example “I think my natural, authentic looks and body shape are also good,” and use the 5-point Likert scoring method (1 = completely disagree, 5 = completely agree). The average score of each question was counted as the total body dissatisfaction score, with a higher score indicating greater dissatisfaction with body image. Cronbach’s α was 0.87.

### Data analysis

All analyses were conducted with SPSS 26.0. Regression analysis is used to explore the influence of social media attention on the internalization of media information and the influence of peer communication on peer comparison. The hierarchical regression method was used to examine the moderating effects of protective filtering on the relationship between social media attention and media information internalization as well as between peer communication and peer comparison. PROCESS macro (Model 4) in SPSS was used to verify the mediating effect of body surveillance between the internalization of social information and body dissatisfaction, as well as between the mediating effect of body surveillance between peer comparison and body dissatisfaction.

## Results

### Preliminary analyses

Pearson’s correlation was conducted (Table 1). The results demonstrate that media attention positively correlates with internalizing media information. Peer communication was also positively correlated with peer comparison. Meanwhile, it is worth noting that some correlations between constructs were higher than the benchmark of 0.6, so a multicollinearity test is needed. The analysis results showed that the highest VIF was 3.18, indicating that multicollinearity is not a significant problem in our dataset [44].

**Table 1:** Descriptive statistics and correlation analysis

Variable	M	SD	Correlations						
			1	2	3	4	5	6	7
1.Media attention	3.86	0.83	-						
2. Internalization of media information	3.64	0.73	0.59^***^	-					
3.Peer communication	3.83	0.88	0.70^***^	0.62^***^	-				
4.Peer comparison	3.76	0.85	0.71^***^	0.69^***^	0.75^***^	-			
5.Body surveillance	3.83	0.83	0.66^***^	0.64^***^	0.67^***^	0.78^***^	-		
6.Body dissatisfaction	3.62	0.93	0.58^***^	0.49^***^	0.58^***^	0.68^***^	0.71^***^	-	
7.Protective filtering	3.82	0.83	0.34^***^	0.26^***^	0.42^***^	0.35^***^	0.33^***^	0.34^***^	-

Note: ***P < 0.001

### Main analyses

#### Test for mediation effect

In Hypothesis 3a, this study proposes that body surveillance was mediators of the link between the internalization of media information and body dissatisfaction. PROCESS macro (Model 4) in SPSS was conducted to test this hypothesis. As evident from Table 2, internalization of media information has a positive prediction on body dissatisfaction, **β** = 0.87, p < 0.001 (Model 1). Internalization of media information was positively linked with body surveillance, **β** = 0.73, p < 0.001 (Model 2), and body surveillance was positively correlated with the extent of body dissatisfaction, **β** = 0.75, p < 0.001 (Model 3).

The indirect effect of internalization of media information on the degree of body dissatisfaction via body surveillance was 0.85 (SE = 0.02, 95% CI = [0.40, 0.45]). The CI did not include zero. Consistent with our hypothesis, the internalization of media information had a significant indirect effect on body dissatisfaction via body surveillance. The results indicates that body surveillance plays a mediating role between the internalization of media information and the level of body dissatisfaction. Therefore, it can be concluded that Hypothesis 3a was considered persuasive.

Hypothesis 3b indicated that body surveillance would play a mediating role between peer comparison and body dissatisfaction. As evident from Table 3, peer comparison was positively correlated with body dissatisfaction, **β** = 0.34, p < 0.001 (Model 1). Peer comparison was positively correlated with body surveillance, **β** = 0.77, p < 0.001 (Model 2), and body surveillance was positively linked to the extent of body dissatisfaction, **β** = 0.53, p < 0.001 (Model 3).

The indirect effect of peer comparison extent on the extent of body dissatisfaction though body surveillance was 0.54 (SE = 0.03, 95% CI = [0.53, 0.58]). The result indicate that peer comparison exerted a significant indirect effect on body dissatisfaction through body surveillance. This indicated body surveillance was mediators of the relationship between peer comparison and the extent of body dissatisfaction. Therefore, it can be concluded that Hypothesis 3b was regarded as valid.

**Table 2:** Testing the mediation effect of internalization of media information on body dissatisfaction

Predictors	Model 1(Body dissatisfacation)		Model 2(Body surveillance)		Model 3(Body dissatisfacation)	
	β	t	β	t	β	t
internalization of media information	0.87	4.22***	0.73	46.90***	0.63	32.32
Body surveillance					0.75	41.63***
R²	0.72		0.64		0.50	
F	1675.21***		2199.18***		1044.64***	

Note: *** p < 0.001

**Table 3:** Testing the mediation effect of peer comparison on body dissatisfaction

Predictors	Model 1(Body dissatisfacation)		Model 2(Body surveillance)		Model 3(Body dissatisfacation)	
	β	t	β	t	β	t
Peer comparison	0.34	15.9***	0.77	71.97***	0.75	52.36
Body surveillance					0.53	24.25***
R²	0.74		0.79		0.68	
F	1918.93***		5170.73***		2741.11***	

Note: *** p < 0.001

#### Testing for Moderation Effect

**Fig. 2:**
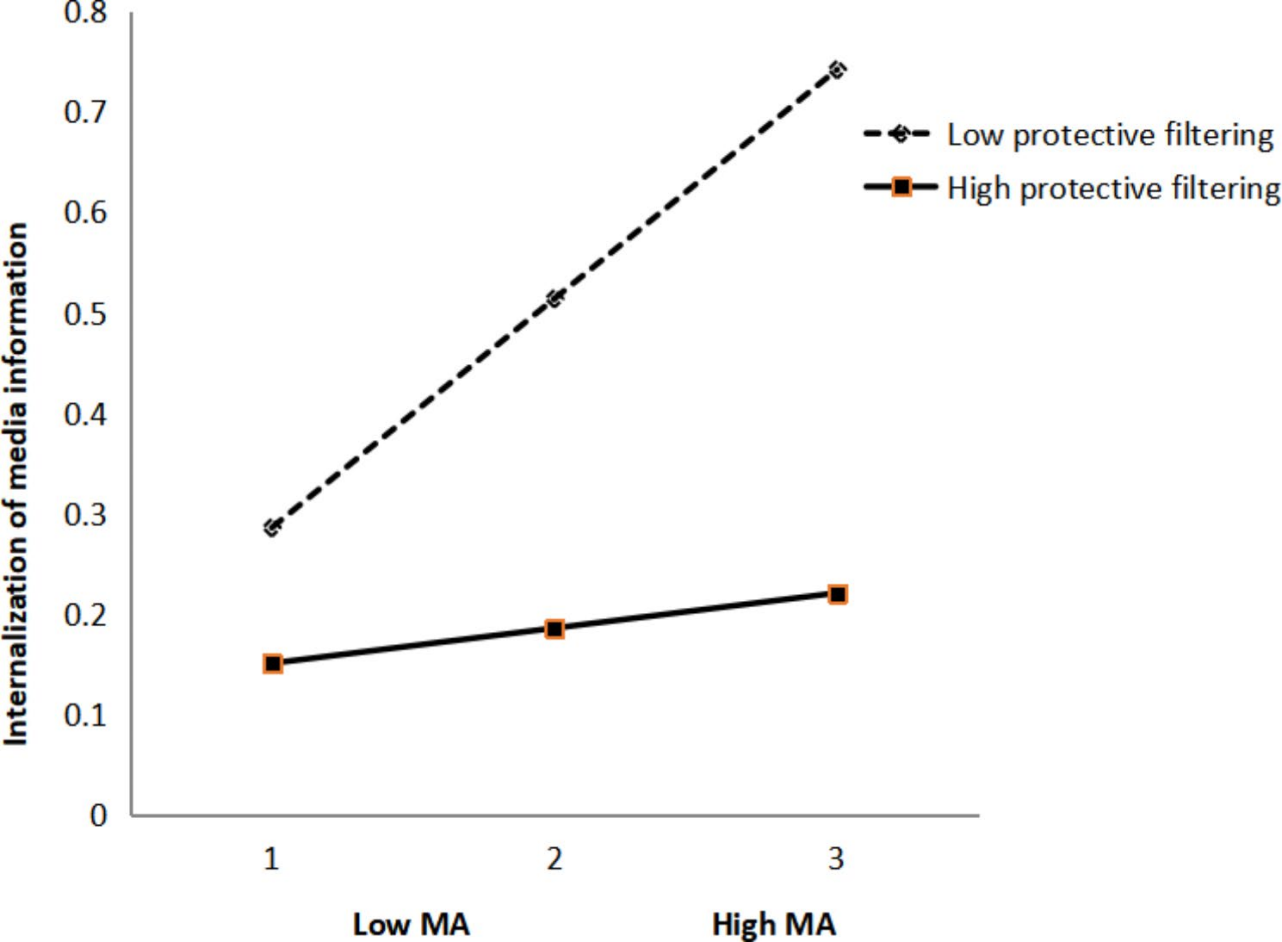
The interaction between media attention and protective filtering on internalization of media information

In Hypothesis 4, we assumed that protective filtering would moderate the relationship between media attention and internalization of media information and moderate the relationship between peer communication and peer comparison. To examine the moderation hypothesis, a hierarchical regression was adopted to analyze the moderating role of protective filtering. First, media attention and internalization of media information were entered. Then, the interaction (media attention × protective filtering) was entered. The results demonstrated significant main effects of media attention and protective filtering on internalizing media information. The effect of the interaction term was also significant, indicating a significant moderating effect of protective filtering on the relationship between media attention and internalization of media information (Table 4). A simple slope test was then adopted, and associated among females with low (− 1 SD) and high (+ 1 SD) levels of protective filtering (Fig. 2). The results demonstrated that the link between media attention and internalization of media information was significant among female with a low level of protective filtering (β = 0.48, p < 0.001) and insignificant among female with a high level of protective filtering (β = 0.07, p > 0.05). These results supported the hypothesis of this study that protective filtering could significantly buffer the positive relationship between media attention and females’ internalization of media information.

**Table 4:** Testing the moderation effect of protective filtering on media attention and internalization of media information

Variables	Model 1	Model 2
	β	β
Media attention(MA)	0.50***	0.42***
Protective filtering(PF)	0.06	0.06**
MA×PF		-0.19**
R2	0.36***	0.40***
ΔR2		0.04***
F	44.53***	36.21***

β values indicate standardized coefficients; MA and PF were mean centered; ** p < 0.01, *** p < 0.001

**Fig. 3:**
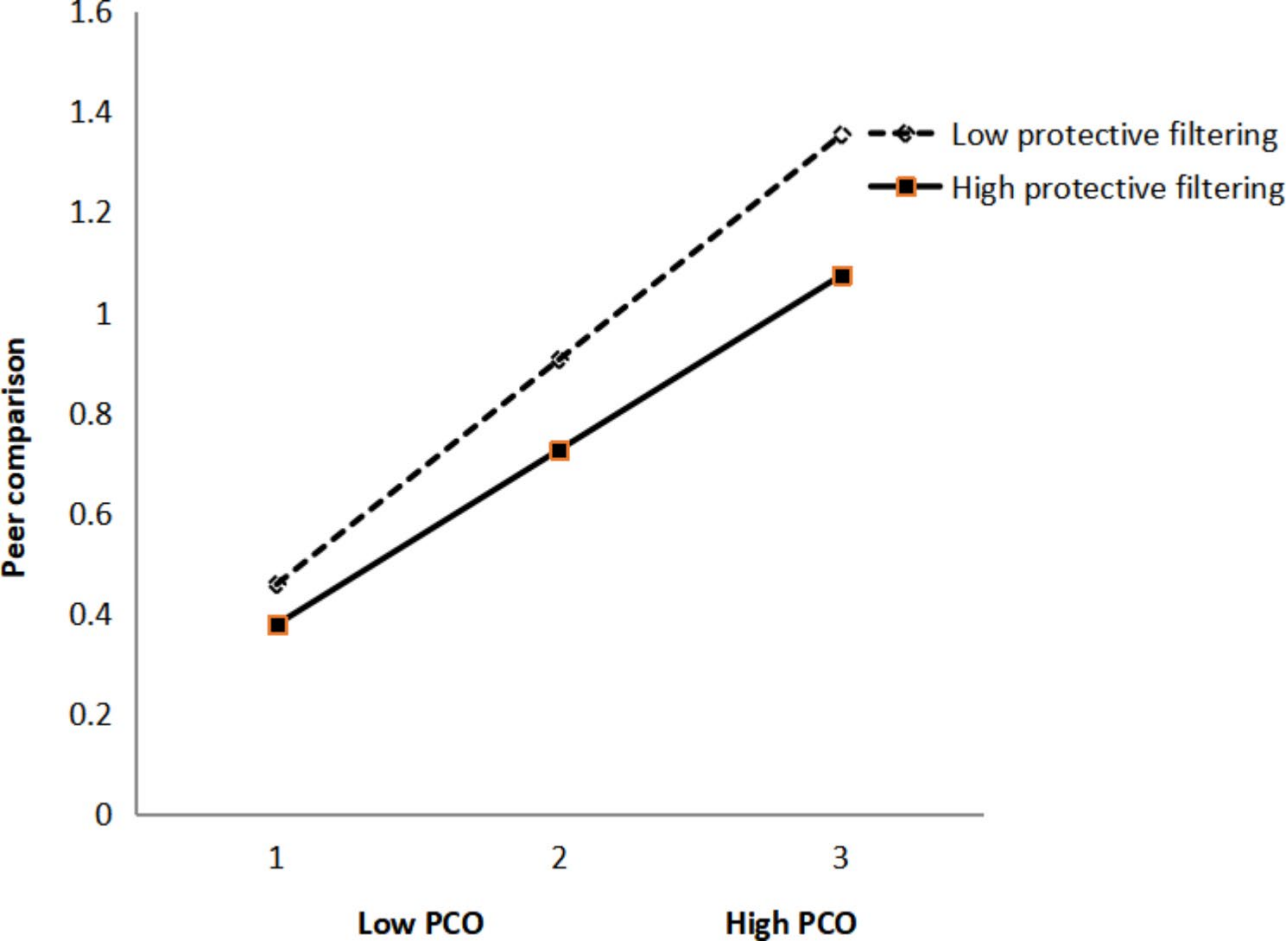
The interaction between peer communication and protective filtering on peer comparison

To examine whether protective filtering moderates the relationship between peer communication and peer comparison, hierarchical regression was adopted to analyze the moderating role of protective filtering. First, peer communication and peer comparison were entered; then, interaction (peer communication × protective filtering) was entered. The results demonstrated significant main effects of peer communication and protective filtering on peer comparison. The effect of the interaction term was also significant, indicating that protective filtering significantly moderates the relationships between peer communication and comparison (Table 5). A simple slope test was then adopted, and associations were conducted among females with low (− 1 SD) and high (+ 1 SD) levels of protective filtering (Fig. 3). The results demonstrated that the relationship between peer communication and peer comparison was significant among female with a low level of protective filtering (β = 0.64, p < 0.001) and insignificant among female with a high level of protective filtering (β = 0.15, p > 0.05). The statistical analysis results above support the hypothesis of this study that protective filtering could significantly buffer the positive relationship between peer communication and peer comparison.

**Table 5:** Testing the moderation effect of protective filtering on peer communication and peer comparison

Variables	Block 1	Block 2
	β	β
Peer communication(PCO)	0.52***	0.50***
Protective filtering(PF)	0.03	0.01**
PCO×PF		-0.05***
R2	0.51***	0.54***
ΔR2		0.03***
F	38.64***	33.32***

β values indicate standardized coefficients; PCO and PF were mean centered; ** p < 0.01, *** p < 0.001

## Discussion

### Overview of findings

Women’s thoughts and feelings about their appearance are complex and affected by many factors. This study combined the theoretical model of sociocultural theory, the objectification theory and the main factor (protective filtering) to examine how media attention and peer communication about appearance-related information affect women’s body image. Several key insights can be inferred from the study’s findings. First, attention to information about beauty-ideal on social media significantly and positively predicted the extent of internalization of beauty ideals, supporting Hypothesis (1) Second, peer communication of appearance-related information is positively related to peer comparison, thus supporting Hypothesis (2) Third, body surveillance played a mediating role between the internalization of beauty ideals and body dissatisfaction as well as peer comparison and body dissatisfaction, supporting Hypotheses 3a and 3b. Finally, and most importantly, protective filtering moderated both the influence of internalization of beauty- ideal information on body satisfaction and the effect of peer comparison on body dissatisfaction, supporting Hypotheses 4a and 4b. Overall, these findings not only support the integrated sociocultural model of body image but also enrich its connotations through constructs from the tripartite influence model, objectification theory and previous studies on protective filtering.

Consistent with our expectations, individuals who paid excessive attention to appearance information in mass media were more likely to have a high degree of internalization. This finding is in conformity with that of Shen et al. [13]. Furthermore, girls who discuss appearance-related information more frequently with their peers were more likely to compare their bodies to others. This finding is consistent with those of Dohnt and Tiggemann [9]. These two findings support the tenets of the theoretical model of sociocultural theory, which emphasizes that pressures from social media and peers, once internalized, can lead to feelings of anxiety about appearance or body shape. Notably, media attention about beauty-ideal and peer communication did not directly predict body dissatisfaction. The internalization of the beauty-ideal information had a chained indirect influence on the media attention and body dissatisfaction. Online media efficiently sets unrealistic standards of ideal beauty and transmit information encouraging women to aspire to it [45]. With frequent exposure to this information, Chinese young adult women with more social orientation characteristics are more easily to internalize and be influenced by these online standards. Second, peer communication has an indirect connection with body dissatisfaction. Peer communication on body appearance, especially talking about one’s appearance, brought self-evaluation motivation and peer comparison. Thus, it is reasonable to speculate that excessive communication on body shape or poor evaluation stimulates young adult women to compare themselves with peers with “good bodies” [25].

Body surveillance has been shown to play a mediating role between the internalization of media attention and peer comparison on body dissatisfaction. This crucial finding provides empirical evidence for the further integration of sociocultural and objectification theory. Firstly, our findings support that the internalization of media information is positive correlated with body dissatisfaction via engaging in greater body surveillance. According to feminist theorists [29], internalization and body surveillance are crucial components of women’s negative experiences of their bodies, and internalization provides an ideal standard for the cultural body. When women compare themselves to the standard but cannot reduce the discrepancy, they may feel bad about their bodies. Meanwhile, consistent with the findings of Vandenbosch and Eggermont’s research [26] on adolescent females, being exposed to sexually objectifying media and internalization of beauty-ideal information may causes females to assess their bodies through an observer’s perspective habitually. Finally, negative evaluations of their body would be generated through body surveillance. Considering China’s traditional patriarchal culture, women’s appearance is often associated with their social and economic benefits [12]. Chinese young adult women with beautiful appearances are likely to have more satisfying marriages and successful careers, which is also consistent with the mainstream ideology advocated by social media. When young adult women internalize information, they create a negative attitude toward their bodies through the mediation of body surveillance.

Our findings also suggested that body surveillance plays a mediating role between peer comparison and body dissatisfaction, which is consistent with the findings of previous research on the correlation between peer effect and body dissatisfaction [24]. As self-objectification theory depicts, self-objectification manifests itself behaviorally as bodily surveillance, which can cause negative influence on psychology [46]. Our study adds further empirical support for aspects of self-objectification theory and verifies the mediating role of body surveillance between peer comparison and body dissatisfaction.

Firstly, the first stages in the mediation model shows that peer comparison is positively correlated with body surveillance, which is consistent with previous findings that there was a significant relationship between peer comparison and body surveillance [47], women who frequently compared themselves to their peers showed higher levels of body surveillance. Secondly, the path from peer comparisons to body dissatisfaction was also significant. This finding can be explained by social comparison theory [48], which indicates that women frequently make appearance-related social comparisons, and that such comparisons are usually upward and horizontal. Compared to upward comparisons with unrealistic images of ideal beauty on social media, compare with peers is more realistic and feasible, because their lifestyles and resources are more similar to women’s own than celebrities. Therefore, horizontal comparisons between peers may be more common in female groups, and these horizontal body comparisons often lead to negative outcomes, such as body dissatisfaction. It is worth noting that there are also studies showing that horizontal comparison with peers is conducive to establish women’s positive feelings about their bodies to a certain extent [49]. Future studies may focus on the positive effects of peer comparison on female body image.

In addition, another core contribution of the study is the moderating effect of protective filtering on two paths (the relationship between media attention and internalization of media information and the link between peer communication and peer comparison). This finding extends the study of Halliwell [34], who used controlled trial to examine whether the protective role of body appreciation can protect women from exposure to information about negative body image in the media. First, the influence of appearance-related media attention on internalization depended on the level of personal protective filtering. When individuals strongly perceive that information will negatively affect their perception of the body and try to reject it, the link between media attention and internalization is weakened. Second, the effect of appearance-related peer communication on peer comparison depended on protective filtering. When a protective filtering approach is applied to the process of communicating about body image with peers, sense of comparison about their bodies will be diminished. Conversations and interactions with peers create a daily context for attending to, constructing, and interpreting information related to appearance or body shape. Protective filtering is defined similarly to systematic processing, which is one of the main information processing strategies in the Heuristic-Systematic Model (HSM) in the field of communication. HSM states that systematic processing means an individual makes a judgment based on thoughtful consideration of concepts and comparing those concepts to information already available [50]. Therefore, it is reasonable to speculate that women with stronger protective filtering are less likely to be negatively influenced by appearance-related peer communication in their rational information processing strategies. To some extent, this finding explains why social media and peer comparison are not always directly related to women’s body image [51].

### Strengths, limitations and future directions

The findings of this study have several theoretical contributions. First of all, it investigates the impact of media attention and peer influence on negative body image and explore its underlying mechanisms. In addition, the study further enriches the body image research of women by combining the sociocultural and self-objectification models and applying them to Chinese context. This design provides us with a deeper understanding of the mechanisms of social media and peer influence on body image. Second, our study considers the protective filtering variable creatively and incorporated this positive body image cognitive strategy into the influencing factor model of negative body image, which not only enriches and expands the tripartite influence model but is also helpful in developing intervention measures to improve body image on the basis of this model.

Moreover, the study has several practical implications. First, by building awareness of surveillance behaviors, as well as structural cognitive and behavioral changes, it may be possible to prevent the internalization of these behaviors into negative body image. Second, more valuable strategies should be adopted to stimulate protective filtering in women. For example, mass media campaigns promoting positive body image can cultivate a social environment that encourages young adult women to emphasize the importance of focusing not only on their physical appearance but also on other valued domains in their lives and spending time with people who are not invested in physical appearance [7].

However, there are some limitations to this study. First, since this is a cross-sectional study, it is not possible to determine the causal relationships between the variables. Experimental methods should be applied to identify complex relationships among related variables. Second, since consumers aged 20 to 29 are the biggest users of social media^1^.the sample age of this study is also mainly concentrated in this age group. Therefore, the results of this study may not apply to women in their forties and older. In addition, the study did not include male group, which has been a growing concern in recent years. Future research may need to enrich the current findings with a more diverse sample in terms of gender, age, socioeconomic status, and ethnicity. Third, our study only verified the moderating effect of protective filtering on the process of internalization of media information and peer comparison, without investigating how protective filtering specifically affects body dissatisfaction. Future research must explore precisely the mechanisms responsible for the influence of protective filtering on negative body image. For example, self-compassion and body appreciation are highly correlated [52], those orientating cognitive processing may contribute to women’s rejection and reconstruction of negative messages from social media and promote a positive view of their body appearance.

## Conclusions

In summary, this study is an important extension of the research on the mechanism of influencing factors of body dissatisfaction among young Chinese women. Meanwhile, it uncovered a positive relationship between media attention and internalization of media information, as well as between peer communication and peer comparison. In addition, the mediating role of body surveillance in the relationship between the internalization of media information and body dissatisfaction, as well as between peer comparison and body dissatisfaction, tested positive. This study provides a brand-new perspective for exploring the innovation of objectified body consciousness and expanding the scope of applicability of the tripartite influence model. Furthermore, our findings indicate that protective filtering can moderate the path of media attention affecting the internalization of media information and the path of peer communication affecting peer comparison, which has important implications for future intervention research and practice.

## Data Availability

The datasets generated during and analyzed during the current study are available from the corresponding author on reasonable request.

## Footnotes

1 What Age Group Uses Social Media the Most? [Aug 2023 Update] (oberlo.com).
